# Behavioral and Neurochemical Characterization of New Mouse Model of Hyperphenylalaninemia

**DOI:** 10.1371/journal.pone.0084697

**Published:** 2013-12-20

**Authors:** Tiziana Pascucci, Giacomo Giacovazzo, Diego Andolina, Alessandra Accoto, Elena Fiori, Rossella Ventura, Cristina Orsini, David Conversi, Claudia Carducci, Vincenzo Leuzzi, Stefano Puglisi-Allegra

**Affiliations:** 1 Dipartimento di Psicologia and Centro “Daniel Bovet”, Sapienza - Università di Roma, Roma, Italy; 2 Fondazione Santa Lucia, IRCCS, Roma, Italy; 3 Dipartimento di Scienze Cliniche Applicate e Biotecnologiche, University of L'Aquila, L'Aquila, Italy; 4 Dipartimento di Medicina sperimentale e Patologia, Sapienza - Università di Roma, Roma, Italy; 5 Dipartimento di Scienze Neurologiche, Psichiatriche e Riabilitative dell'Età Evolutiva, Università di Roma, Roma, Italy; Rikagaku Kenkyūsho Brain Science Institute, Japan

## Abstract

Hyperphenylalaninemia (HPA) refers to all clinical conditions characterized by increased amounts of phenylalanine (PHE) in blood and other tissues. According to their blood PHE concentrations under a free diet, hyperphenylalaninemic patients are commonly classified into phenotypic subtypes: classical phenylketonuria (PKU) (PHE > 1200 µM/L), mild PKU (PHE 600-1200 µM/L) and persistent HPA (PHE 120-600 µM/L) (normal blood PHE < 120 µM/L). The current treatment for hyperphenylalaninemic patients is aimed to keep blood PHE levels within the safe range of 120-360 µM/L through a PHE-restricted diet, difficult to achieve. If untreated, classical PKU presents variable neurological and mental impairment. However, even mildly elevated blood PHE levels, due to a bad compliance to dietary treatment, produce cognitive deficits involving the prefrontal cortical areas, extremely sensible to PHE-induced disturbances. The development of animal models of different degrees of HPA is a useful tool for identifying the metabolic mechanisms underlying cognitive deficits induced by PHE. In this paper we analyzed the behavioral and biochemical phenotypes of different forms of HPA (control, mild-HPA, mild-PKU and classic-PKU), developed on the base of plasma PHE concentrations. Our results demonstrated that mice with different forms of HPA present different phenotypes, characterized by increasing severity of behavioral symptoms and brain aminergic deficits moving from mild HPA to classical PKU forms. In addition, our data identify preFrontal cortex and amygdala as the most affected brain areas and confirm the highest susceptibility of brain serotonin metabolism to mildly elevated blood PHE.

## Introduction

Hyperphenylalaninemia (HPA) refers to all clinical conditions characterized by elevated blood phenylalanine (PHE) concentrations due to impairment of the enzyme PHE hydroxylase (PAH). Hyperphenylalaninemic patients are commonly classified into phenotypic subtypes according to their blood PHE concentrations under a free diet: classical PKU (PHE > 1200 µM), mild PKU (PHE 600-1200 µM) and persistent HPA (PHE 120-600 µM) (normal blood PHE < 120 µM). The recommended treatment for all HPA forms is a PHE-restricted diet that should be continued as long as possible. Nevertheless, the compliance with dietary treatment is difficult to achieve and, from adolescence, 60 to 80% of the patients have partially or totally abandoned the treatment [1], producing significant increase of PHE blood levels that results in a relevant risk of cognitive skills, particularly in frontal/executive functions. These functions are associated with the preFrontal Cortex (pFC) that, more than other cerebral regions, appears to be extremely sensible to PHE-induced damages. In fact, deficits in working memory and executive functions are reported in children with higher current PHE concentrations and in adults with early-treated PKU after they relaxed or stopped the diet [2,3,4,5,6,7,8,9]. 

The development of a genetic murine model of PKU has raised the hope to elucidate the mechanism by which PHE damages cortical functioning and to develop alternative treatments. PAH^enu2^ mice (ENU2), created by chemically induced genetic mutation, are characterized by a biochemical phenotype that closely resembles untreated human PKU, being characterized by reduced PAH activity, PHE plasma levels 10-20 times greater that those of normal littermates [10,11,12,13,14] and deficits of biogenic amines [15,16,17,18,19,20]. If ENU2 mice are the most commonly used model of PKU, no valid murine models for different degrees of HPA are available, to our knowledge.

Here, we present behavioral and biochemical characterization of mouse model of different forms of HPA, showing the effects of increasing blood PHE levels. 

## Materials and Methods

### Animals

All animals were handled in strict accordance with good animal practice as defined by the relevant national and local animal welfare bodies. All experiments of this study were approved by the ethics committee of the Italian Ministry of Health and therefore conducted under license/approval ID #: 10/2011-B, according with Italian regulations on the use of animals for research (legislation DL 116/92) and NIH guidelines on animal care.

Adult homozygous and heterozygous male ENU2 mice and male mice of the background BTBR strain were issued from heterozygous mating. Genetic characterization was performed on DNA prepared from tail tissue using the Easy DNA Kit (Invitrogen, Carlsbad, CA, USA). The enu2 mutation was detected after PCR amplification of exon 7 of the Pah gene and digestion with BsmAI restriction enzyme (NEB, USA) as described [17]. Further studies have shown that the ENU2 mouse is characterized by a radical mutation located on exon 7, a gene region where serious mutations are common in humans, and by a phenotypic profile strikingly similar to severe human PKU [10,11,12,13,14].

At postnatal day 28, animals (sex matched) were housed 2-4 per standard breeding cage with food and water ad libitum on a 12:12h dark: light cycle (light on 07.00 am - 07.00 pm h). Experiments started when animals reached 8 weeks of age. Mice were housed in appropriate animal facilities. Every effort was made to alleviate animal discomfort and cervical dislocation was applied as the appropriate method of sacrifice.

Different forms of HPA were obtained as follows: control mice (Cntr): BTBR mice; mild-HPA (m-HPA): heterozygous mice; mild-PKU (m-PKU): ENU2 mice maintained with a PHE-free diet and receiving PHE in drinking water (2 gr per liter), and classical PKU (c-PKU): ENU2 mice.

### Drugs

PHE-free diet were purchased from Research Diet Inc. (New Brunswick, USA). L-PHE dissolved in water were purchased from Sigma-Aldrich (St. Luis, MO, USA).

### Behavioral assay

All tests were conducted in a sound-attenuated cubicle, and videotaped by means of a camera placed within the cubicle and connected to a recorder placed outside the cubicle. Video-based EthoVision System (Noldus, The Netherlands) was used to record, collect and analyze data. Details of apparatus and procedures have been previously described [13,15].

Four groups of male mice (Cntr, m-HPA, m-PKU and c-PKU; N=8 for group) were submitted to Elevated Plus Maze, Open Field Test and Object Recognition Test, in this order.

Two additional groups of male mice (Cntr, m-HPA; N=10 for group) were subjected to Delayed Alternation Test. Three mice (2 m-HPA and 1 Cntr) did not reach the learning criterion and were excluded from the experiment (Cntr, N=9; m-HPA; N=8).

### Elevated Plus Maze (EPM)

Mice were individually tested in a single 6-min session in the EPM. The total number of entries in arms, the percentage of entries in the open arms (open entries/open + closed x 100), and the percentage of time spent in the open arms (time in open/open + closed x 100) were recorded. One-way ANOVAs, followed by post-hoc Duncan’s test for multiple comparisons, were used for statistical analysis of the effects of groups (Cntr, m-HPA, m-PKUand c-PKU; N=8 for group) on all parameters.

### Open Field Test (OFT)

The OFT represent the first session of Object Recognition Test. Apparatus was a circular open field, 60 cm in diameter and 20 cm in height. During OFT, the mouse was introduced in a specific sector of the empty open field and left to explore the apparatus for 5 minutes. Distance moved (cm), velocity (cm/sec) and percentage of time spent in the center ([sec center/300sec]x100) were recorded. One-way ANOVAs, followed by post-hoc Duncan’s test, were used for statistical analysis of the effects of group (Cntr, m-HPA, m-PKUand c-PKU; N=8 for group) on all parameters.

### Object Recognition Test (ORT)

Each mouse was individually submitted to three successive sessions (Open Field, Pre-Test and Test sessions). At the end of each session, the subject was returned to its home cage for 3 min, and the apparatus was cleaned with a solution of water and ethanol. During Pre-Test session (5 min), the total time spent exploring objects was analysed by one-way ANOVA with group as factor (four levels: Cntr, m-HPA, m-PKUand c-PKU; N=8 for group), followed by post-hoc Duncan’s test. Object recognition was evaluated in Test session (5 min) by comparison of novel vs. familiar object exploration. The position of the objects in the Test session was balanced between mice and undesirable side effect controlled. The total time spent exploring each object on the test session was evaluated by two-way ANOVA for repeated measures (group, four levels: Cntr, m-HPA, m-PKUand c-PKU as between factor and ‘object’: two levels A3 and B, as within factor), with p<0.05 as significance criteria. Simple effect analysis of the factor ‘object’ was also evaluated within each group.

### Delayed Alternation Test (DAT)

The T-maze apparatus (gray Polyvinyl Chloride, PVC) consisted of a central stem (height 20, length 74 and width 10 cm) with a start box (the first 10 cm of the stem) and two arms (height 20, length 15 and width 10 cm). Guillotine doors separated the start compartment and each goal arm that were differentiated by graphic patterns (horizontal or vertical black/white stripes). Complete entrance into the arm was required in order to enable mice to reach the food cup located at the end. Low intensity diffuse illumination was provided above the apparatus. The apparatus was cleaned with a solution of ethanol (10%) and distilled water between trials and after each individuals animals. Kellogg’s honey cheerios was chosen as reward due to its low content in PHE. Throughout the experiment animals had limited access to food (two hours immediately after each daily session).

Training procedure consisted of a forced alternation and a free choice phase. Training of the forced alternation phase consisted in six sessions of forced choice (6 trials x day). After short confinement in the start box (10 sec), the door was lifted and mice were forced to enter the only accessible arm, as access to the opposite arm was blocked. The baited compartment was alternated on trials, thus forcing mice to enter alternatively the right or left arm.

In the free choice training mice were required to freely alternate between right and left arms in order to obtain a food reward. This phase consisted in daily sessions of 11 trials after the sample trial. On the first trial both goal arms were baited and mice’s choice set the sample. On the subsequent 11 trials the only baited arm was the opposite of the last one where reward was found (independently by the last chosen arm). A trial ended when mice reached the food reward or entered in the unbaited arm. Between trials mice were confined in the start box (10 sec). The criterion for learning of the non-matching to sample rule was defined as 80% correct entries averaged over three consecutive days, which sets the baseline (bsl) for the delay phase. A maximum of 27 sessions was allowed to reach the criterion. Once the criterion was reached, delayed alternation was tested by interposing 30, 40 and 60 sec delay between trials. Each delay was used for 2 consecutive days. Percentage of correct responses was evaluated by two-way ANOVA for repeated measures (group, two levels: Cntr and m-HPA as between factor, N=8-9 for group; and delays, four levels: 0, 30, 40, 60 sec as within factor), with p<0.05 as significance criteria.

### Neurochemical assay

Four groups of naïve male mice (Cntr, m-HPA, m-PKU and c-PKU; N=6 for group) were used to verify HPA status.

For peripheral PHE assay, blood drop was collected on filter paper and dried blood spots were assessed by ESI-MS/MS [21]. One-way ANOVA, followed by post-hoc Duncan’s test, was used for statistical analysis with p<0.05 as significance criteria.

Four groups of naïve male mice (Cntr, m-HPA, m-PKU and c-PKU; N=12 for group) were used to analyze cerebral aminergic metabolism. In particular, for analysis of cerebral levels of biogenic amines and their metabolites (3-4- Dihydroxyphenylacetic acid, DOPAC; homovanillic acid, HVA; 3-methoxy-4 hydroxyphenylethyleneglycol (MHPG) and 5-hydroxyindoleacetic acid, 5-HIAA), brains were collected postmortem and punches of pFC, Cingulate Cortex (CgC), Nucleus Accumbens (NAc), Caudate Putamen (CP), Hippocampus (HIP) and Amygdala (AMY) were obtained as previously reported [20]. Punches were weighed and homogenized in 0.05 M HClO4. The homogenates were centrifuged at 14000 rpm for 20 min at 4° C and supernatant were transferred to HPLC system (Alliance, Waters Corporation, Milford, MA) coupled with a coulometric detector (model 5200 Coulochem II; ESA, Chelmsford, MA). The potentials were set at +450 mV and +100 mV at the analytical and conditioning cell, respectively. The columns, a Nova-Pack Phenyl column (3.9 x 150mm) and a sentry GuardNova-Pack Phenyl (3.9 x 20mm) maintained at 28 °C, were purchased from Waters Corporation. The flow pack rate was 1 ml/min. The mobile Phase consisted of 3% methanol in 0.1M Na-phosphate buffer pH 3, 0.1mM, Na2 EDTA and 0.5mM 1-octane sulphonic acid Na salt (Aldrich).

One-way ANOVAs, followed by post-hoc Duncan’s test, were used for statistical analysis of the effects of groups (Cntr, m-HPA, m-PKUand c-PKU; N=12 for group) for each amine and metabolite (ng/g wet weight) within each brain area, with p<0.05 as significance criteria.

## Results

### Assessment of HPA status

One-way ANOVA revealed a significant effect of group for blood PHE levels (F(3,20) = 70,29 p<.0001). Post-hoc analysis indicated significant difference among groups (Cntr: 66.00 + 6.38 µM/L; m-HPA: 200.77 + 17.03 µM/L; m-PKU: 712.58 + 145.18 µM/L; c-PKU: 1473.22 + 41.41 µM/L).

**Figure 1:**
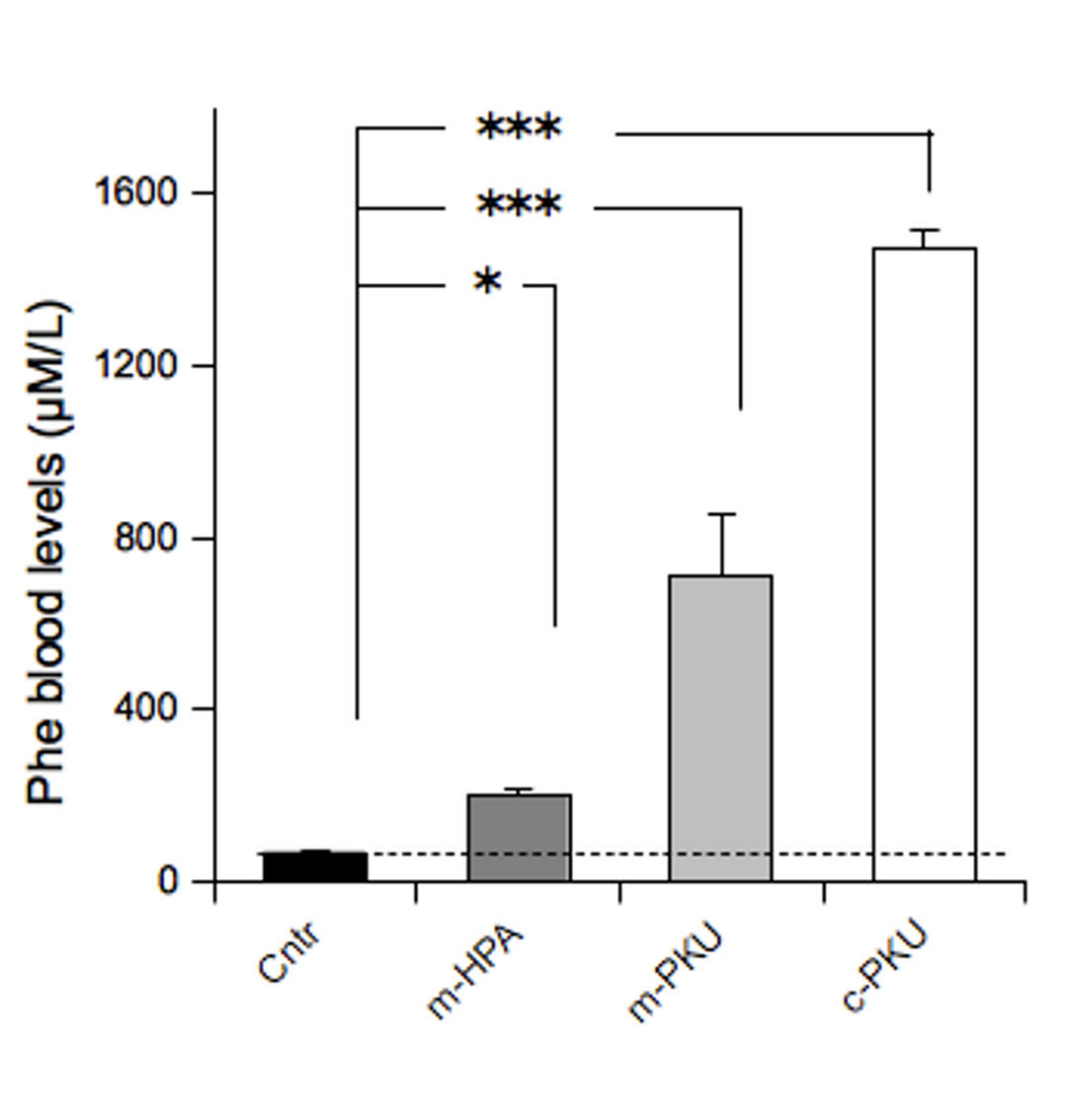
Blood levels of PHE in different forms of HPA. Values are expressed as means + S.E.M. N=6 for group. *,*** p<0.05, 0.001 from Cntr mice.

These results confirm the development of murine model of different degrees of human HPA (Figure 1).

### Assessment of emotional reactivity

**Figure 2:**
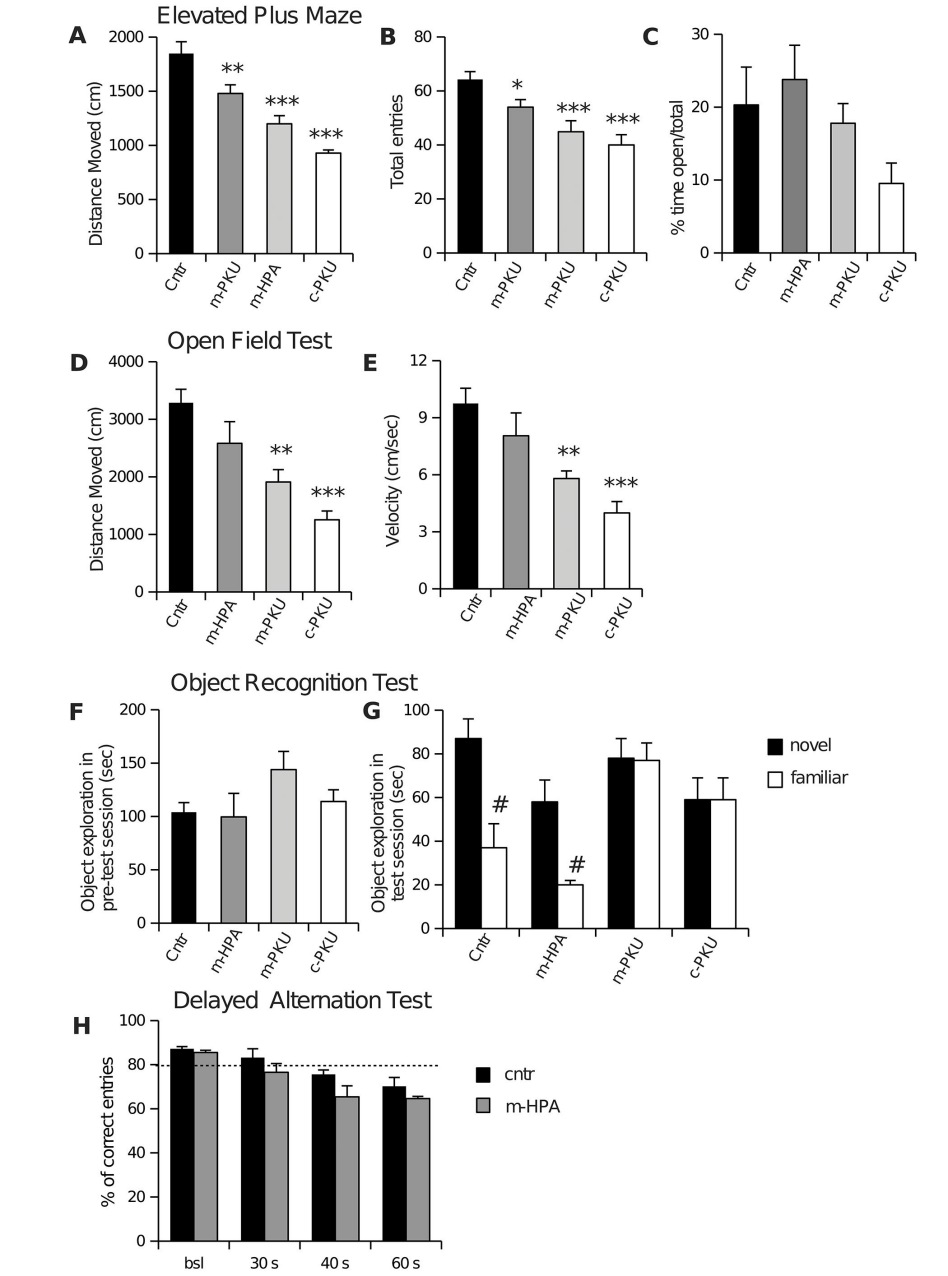
Behaviors of HPA mice. Elevated Plus Maze (A, B, C). Compared to Cntr mice, all other groups showed decrease in distance moved (B) and total entries (A), but no significant differences in the percent of time and entries in open arms (B). Open Field Test (D, E). Compared to Cntr mice, m-PKU and c-PKU showed significant decrease in distance moved (D) and velocity (E). Object Recognition Test (F, G). Results are expressed as mean time spent exploring the two identical objects during pre-test session (F) and as time spent exploring either the novel or the familiar object during test session (G). Only Cntr and m-HPA mice showed object recognition ability, spending more time exploring novel object that the familiar one. Delayed Alternation Task (H). The performance below the criterion level (dotted line) was evident in the m-HPA mice after the shortest delay (30 sec). All data are are expressed as means + S.E.M. *,*** p<0.05, 0.001 vs. all other groups; # p<0.05 vs. familiar object.

In Figure 2 (A, B, C) are reported data from the EPM. One-way ANOVAs revealed significant effect of group only for distance moved (F(3,28) = 25.06; p<0.001) and total entries (F(3,28) = 9.50; p<0.001). Compared to Cntr mice, all other groups showed decrease in distance moved and total entries. One-way ANOVA does not revealed differences between groups in the percent of time and entries in open arms, although data showed a tendency to reduce time in open arms ranging from m-HPA to c-PKU groups.

These results indicate that blood PHE levels interfere mainly with motor activity in EPM.

### Assessment of locomotor activity

In Figure 2 (D, E) are reported data from OFT. One-way ANOVAs revealed significant effect of group for both distance moved (F_(3,28)_ = 11.43; p<0.001) and velocity (F_(3,28)_ = 9.04; p<0.001). Compared to Cntr mice, m-PKU and c-PKU showed significant decrease in distance moved and velocity, although there was a non-significant decrease also in m-HPA mice. No significant difference was evident between groups in percentage of time spent in the center of arena.

These results confirm that HPA status interfere with locomotor activity.

### Assessment of novel object exploration

In Figure 2 (F, G) are reported data from the ORT. The ORT is a variant for rodents of the delayed non-matching to sample task. It is a non-associative test that does not require reinforcement and exploit spontaneous preference of mice for novelty [13]. Statistical analysis excluded preferences for object location. One-way ANOVA does not reveal significant difference among groups for the time spent exploring the objects during training session, suggesting that all mice show normal reactivity to stimuli presentation (Figure 2, F). Two-way ANOVA analysis performed on test session revealed significant interaction between factors ‘object’ and group (F_3,28_ = 4.78, p<0.01). Post-hoc analysis revealed significant difference in the time spent exploring the two objects (novel vs familiar) only in Cntr and HPA groups that spent more time in exploring the new object than the familiar object. Conversely, m-PKU and c-PKU mice do not display the typical spontaneous preference of rodents for novel stimuli, showing similar exploration between novel and familiar stimuli (Figure 2, G) .

Results obtained in the ORT confirm cognitive deficits previously reported in c-PKU mice [13,15] and demonstrate that mild blood PHE levels are unable to impair ability to coding and retrieving object information.

Since ORT was unable to highlight cognitive impairments in mildly hyperphenylalaninemic mice, Cntr and HPA groups were submitted to more stringent cognitive test, involving executive functions.

### Assessment of performances in DAT

In Figure 2 (H) are shown the results of DAT. This task is sensitive to working memory impairment pFC-related [22,23,24,25].

Two-way analysis of the delayed performance revealed significant effects of group (F_1,45_ = 11.60, p<0.01) and delay (F_3,45_ = 23.37, p<0.001) indicating that Cntr mice were overall different from m-HPA mice and that performances of both Cntr and m-HPA mice were affected by the delays. Although, no significant interaction group x delay was found, post-hoc analysis revealed that the 40 seconds delayed performance of Cntr mice was significantly different from the corresponding delayed performance of m-HPA mice (t=2.197 p=0.0352), thus strongly supporting strain differences indicated by significant main effects. Moreover, decreased working memory function in the delayed alternation task is mainly revealed by performance below the criterion level (80% correct entries averaged over three consecutive days), which is indicative of the chance role in entering one arm in a dichotomic choice. The performance below the criterion level was evident only in the m-HPA mice after the shortest delay (30 sec) (as shown in Figure 2, H), thus suggesting in this group higher vulnerability of pFC functioning.

### Neurochemical assessment of brain regions

**Figure 3:**
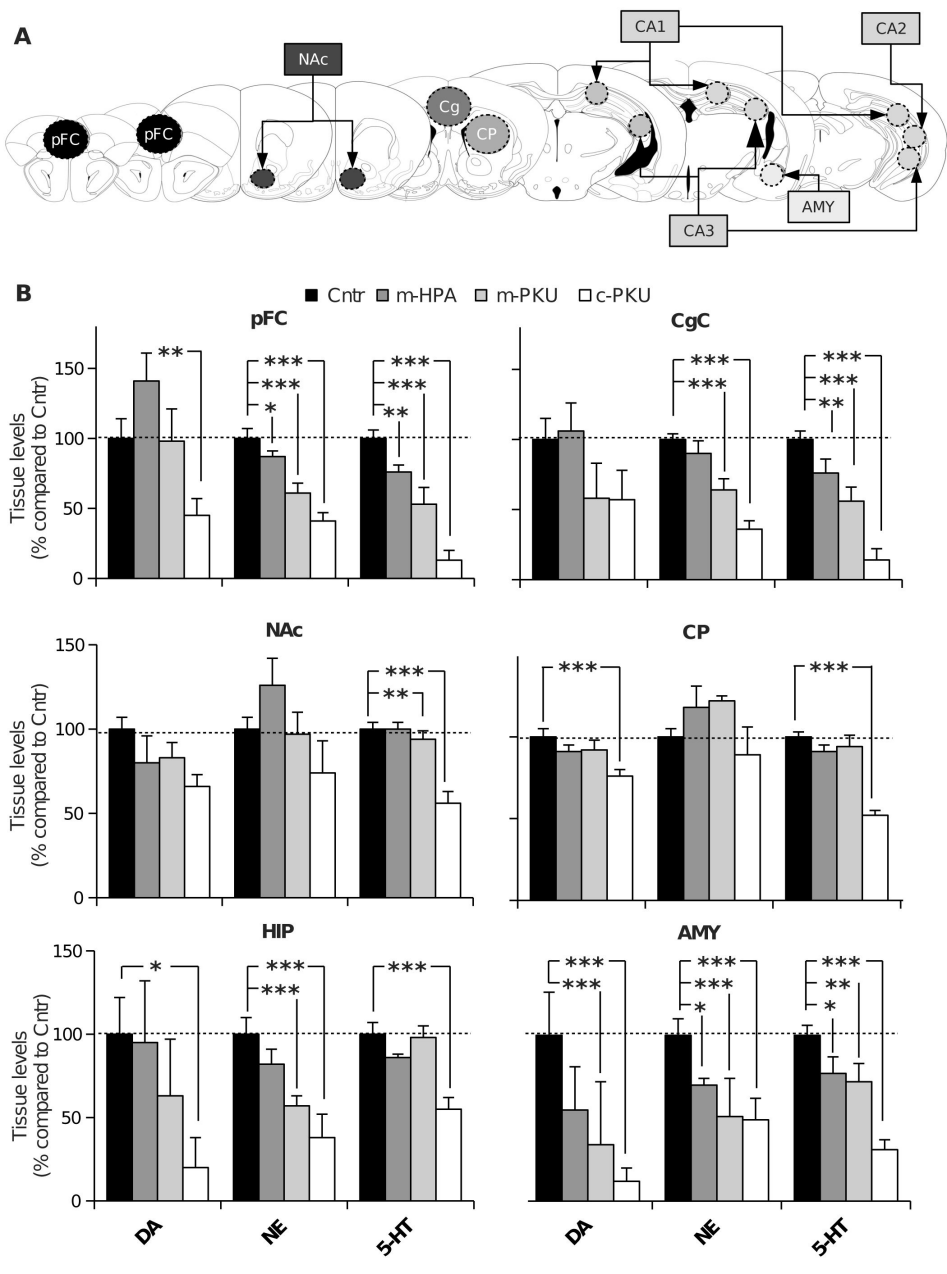
Brain biogenic amine levels in different forms of HPA. (A) Schematic rapresentation of brain areas. (B) Tissue levels of biogenic amines in: preFrontal Cortex (pFC), Cingulate Cortex (CgC), Nucleus Accumbens (NAc), Caudate Putamen (CP), Hippocampus (HIP; CA1, CA2 and CA3 fields) and Amygdala (AMY). Data are reported as percentage + S.E.M compared to Contr group (N=12 for group). Moving from mild HPA to classical PKU forms, data showed progressive impairment in the brain aminergic metabolism. With respect to amine, 5-HT appeared to be most affected and DA the least affected by high blood PHE levels. With respect to brain area, pFC and AMY resulted to be the most susceptible to increased blood PHE levels. *,***** p<0.05, 0.01, 0.001 from Cntr mice.

Results are reported in Table 1 and Figure 3 (B). In Table 1, data are reported as mean concentrations of neurotransmitters and metabolites, whereas in Figure 3 concentrations of neurotransmitters are reported as percentage of average control values. One-way ANOVAs revealed a significant effect of group (four levels: Cntr, m-HPA, m-PKU and c-PKU; N=12 for group) for almost all amines and metabolites in all investigated brain areas, with the exception of DA in CgC, NAc and HIP, DOPAC and HVA in pFC and NE in NAc and CP.

**Table 1:** Tissue levels of biogenic amines and their metabolites (ng/g wet weight) in different brain areas of HPA mice.

		**DA**	**Dopac**	**Hva**	**NE**	**Mhpg**	**5-HT**	**5-hiaa**
**m-pFC**	Cntr	**118.0 + 16.8**	42.9 + 9.9	49.4 + 4.3	**664.4 + 44.6**	**55.0 + 4.4**	**1366.8 + 77.3**	**248.9 + 25.5**
	m-HPA	**165.9 + 33.4**	37.3 + 3.8	48.3 + 1.7	**575.0 + 23.6^a,c,d^**	**49.2 + 3.0^a^,^d^**	**1040.7 + 48.8^a,c,d^**	**230.4 + 22.5^a^,^d^**
	m-PKU	**115.9 + 26.5**	30.3 + 10.4	50.0 + 6.4	**403.0 + 26.6^a,b,d^**	**35.1 + 1.6^a,b,d^**	**725.9 + 88.2^a,b,d^**	**175.1 + 20.6^d^**
	c-PKU	**53.3 + 6.2^b^**	18.7 + 3.2	41.4 + 4.5	**273.2 + 15.3^a,b,c^**	**25.5 + 2.8^a,b,c^**	**175.3 + 11.7^a,b,c^**	**70.8 + 17.3^a,b,c^**
**CgC**	Cntr	332.3 + 90.9	**66.6 + 5.0**	**56.2 + 5.6**	**647.3 + 28.2**	**64.8 + 4.8**	**733.5 + 47.3**	**179.6 + 10.6**
	m-HPA	353.1 + 38.59	**67.6+ 5.3^c^,^d^**	**64.1 + 9.2^c^,^d^**	**583.6 + 52.0^c^,^d^**	**64.2 + 5.4^c^,^d^**	**559.3 + 45.4^a,c,d^**	**142.0 + 3.7^a,c,d^**
	m-PKU	192.7 + 56.18	**47.6 + 2.7^a^,^b^**	**41.0 + 4.8^b^**	**413.0 + 31.8^a,b,d^**	**33.3 + 3.3^b^,^d^**	**409.2 + 42.0^a,b,d^**	**96.8 + 9.7^a,b,d^**
	c-PKU	188.3 + 59.6	**42.4 + 2.5^a^,^b^**	**34.4 + 2.53^a^,^b^**	**234.7 + 14.1^a,b,c^**	**23.1 + 1.5^a^,^b^**	**99.9 + 7.9^a,b,c^**	**31.2 + 1.4^a,b,c^**
**NAc**	Cntr	12500.1 + 862.7	**1059.9 + 63.0**	**848.6 + 54.2**	1409.4 + 103.5	**85.0 + 1.5**	**1855.5 + 77.3**	**1255.1 + 24.7**
	m-HPA	9942.1 + 1555.3	**882.2 + 99.8^c,^**	**784.5 + 49.9^d^**	1773.2 + 278.7	**96.0 + 7.5^c^,^d^**	**1846.9 + 82.5^d^**	**1101.3 + 49.1^d^**
	m-PKU	10374.7 + 929.5	**680.1 + 66.8^a^,^b^**	**758.0 + 47.7**	1365.9 + 172.2	**67.5 + 4.5^a,b,d^**	**1739.4 + 79.8^d^**	**956.1 + 117.7^a^,^d^**
	c-PKU	8289.1 + 555.3	**519.4 + 16.5^a^,^b^**	**646.6 + 16.3^a^,^b^**	1037.2 + 200.5	**45.0 + 5.5^a,b,c^**	**1029.8 + 71.1^a,b,c^**	**270.2 + 11.0^a,b,c^**
**CP**	Cntr	**21626.9 + 1109.6**	**1202.1 + 80.2**	**1388.5 + 57.2**	92.5 + 4.8	**14.4 + 1.8**	**770.0 + 26.2**	**698.4 + 39.4**
	m-HPA	**19672.6 + 856.8^d^**	**1054.1 + 61.4^d^**	**1234.6 + 81.2^d^**	109.1 + 14.4	**10.3 + 0.9^a,c,d^**	**697.7 + 24.8^a^**	**532.9 + 26.7^a^,^d^**
	m-PKU	**19967.0 + 1267.8^d^**	**885.9 + 76.6^a^,^d^**	**1250.9 + 81.2^d^**	112.0 + 3.8	**5.4 + 0.3^a ,b^**	**725.6 + 52.4^d^**	**525.7 + 71.5^a^,^d^**
	c-PKU	**16368.7 + 438.4^a,b,c^**	**629.1 + 24.2^a,b,c^**	**1010.4 + 51.8^a,b,c^**	82.4 + 14.2	**4.5 + 0.6^a^,^b^**	**400.9 + 11.6^a,b,c^**	**128.7 + 22.5^a,b,c^**
**HIPP**	Cntr	695.3 + 150.0	**70.9 + 10.7**	**105.2 + 16.2**	**500.5 + 49.2**	**13.5 + 0.6**	**1641.8 + 111.4**	**621.6 + 50.0**
	m-HPA	662.7 + 243.1	**61.4 + 10.0^c^,^d^**	**90.5 + 20.0^d^**	**408.4 + 37.5^c^,^d^**	**11.6 + 0.7^c^,^d^**	**1406.1 + 33.3^d^**	**489.7 + 26.1^a^,^d^**
	m-PKU	435.6 + 150.3	**30.2 + 5.5^a^,^b^**	**65.4 + 11.4**	**285.7 + 18.4^a^,^b^**	**8.9 + 0.7^a,b,d^**	**1608.8 + 106.1^d^**	**434.3 + 42.7^a^,^d^**
	c-PKU	384.2 + 25.0**^a^**	**22.2 + 2.2^a^,^b^**	**44.0 + 5.0^a^,^b^**	**191.3 + 25.9^a^,^b^**	**6.2 + 0.9^a,b,c^**	**900.2 + 58.6^a,b,c^**	**203.7+ 11.9^a,b,c^**
**AMY**	Cntr	**814.6 + 215.7**	**208.5 + 18.8**	**231.4 + 39.4**	**743.2 + 75.6**	**38.5 + 3.2**	**1955.7 + 124.9**	**846.6 + 100.4**
	m-HPA	**445.8 + 117.4**	**157.2 + 14.6^a,c,d^**	**131.8 + 16.8^a^,^d^**	**515.6 + 20.2^a^**	**29.3 + 2.1^a,c,d^**	**1545.5 + 152.4^a^,^d^**	**497.7 + 62.4^a^,^d^**
	m-PKU	**275.5 + 104.1^a^**	**71.5 + 8.7^a^,^b^**	**108.7 + 18.1^a^**	**377.8 + 85.8^a^**	**19.9 + 3.7^a^,^b^**	**1416.3 + 152.4^a^,^d^**	**479.9 + 98.0^a^**,**^d^**
	c-PKU	**95.7 + 7.9^a^**	**42.4 + 3.4^a^,^b^**	**58.4 + 3.3^a^,^b^**	**361.1 + 46.8^a^**	**13.4 + 1.6^a^,^b^**	**602.1 + 35.1^a,b,d^**	**209.5 + 39.4^a,b,c^**

Values are expressed as means + S.E.M. N = 12 per group.

In bold font is indicated significant effect of group x amine or metabolite.

**^a^** p<0.05, vs Cntr group; **^b^** p<0.05, vs m-HPA group; **^c^** p<0.05, vs m-PKU group; **^d^** p<0.05, vs c-PKU group

In general, neurochemical data showed progressive impairment in the brain aminergic metabolism, moving from mild HPA to classical PKU forms (Fig. 3, B; Table 1). In particular, the levels of 5-HT and of its metabolite 5-HIAA were significantly reduced in all experimental groups regardless of the brain area. NE levels were significantly and progressively reduced in the pFC, CgC, AMY and HIP, although levels of NE metabolite MHPG were significantly reduced in all brain areas. Levels of DA were partially reduced in pFC, CP, HIP and AMY, and its metabolites in all brain area with the exception of pFC.

## Discussion

### The development and characterization of mice with different degrees of HPA is the major goal of the present study.

Consolidated evidence indicates that even mildly elevated blood PHE levels produce cognitive deficits.The most generally accepted hypothesis is that elevated blood PHE concentrations pervade the brain, producing neurotoxic effects [26]. However, the pathophysiologic mechanism by which PHE produces cognitive dysfunction need to be thoroughly investigated. The mouse model of HPAs here proposed provides a good opportunity to assess cognitive consequences of progressive increases of blood PHE concentrations and their correlation with brain metabolic alterations. 

Because hyperphenylalaninemic patients are commonly classified in three forms (classical PKU, PHE > 1200 µM; mild PKU, PHE 600-1200 µM; and persistent HPA, PHE 120-600 µM; normal blood PHE < 120 µM) we modeled these forms in the mouse. In particular, in addition to ENU2 mice (c-PKU group), that represent the best model of classical PKU, we identified heterozygous Pah^Enu2^ mice as a good model for HPA condition and we restricted PHE intake in ENU2 mice in order to maintain a constant mild PKU status.

Behavioral analysis confirm severe deficits in c-PKU group [27], characterized by locomotor disturbances and impairments in recognition of novel objects. m-PKU mice maintain a altered behavioral profile, although locomotor disturbance results reduced in comparison with c-PKU mice. Finally, m-HPA mice, with blood PHE concentrations comparable to patients with persistent HPA, resulted in moderate impairments of locomotor activity and normal ability to discriminate between old and novel objects. For this reason, and on the base of clinical data showing that mildly elevated blood PHE levels induce deficits particularly in executive abilities [3,4,5,6,7,8,9,28,29,30], we submitted m-HPA mice to the DAT, a test strongly associated with medial pFC functions in rodents, wherein m-HPA mice showed a decreased performance after the shortest delay, suggesting a vulnerability of pFC functioning.

Clinical and preclinical neurochemical studies showed reduced aminergic metabolism in brain [31] and cerebrospinal fluid [32,33,34] of PKU patients and in PKU mouse brain [16,17,18,19,20]. In our study, neurochemical analysis revealed PHE-induced impairment of cerebral aminergic systems that exacerbates ranging from mild HPA to classical PKU forms. The dramatic reduction of brain amines content observed in c-PKU mice is consistent with our previous data [20]. Although amine deficits in c-PKU mice appear to involve all the amines and all the brain areas, 5-HT appeared to be most affected and DA the least affected amine, and pFC and AMY the most susceptible areas. Regarding m-PKU mice, neurochemical analysis showed aminergic impairment similar to c-PKU group, but less dramatic. Finally, concerning m-HPA group (i.e. heterozygous Pah^Enu2^ mice), neurochemical analysis showed restricted aminergic deficits, in particular for 5-HT, NE and their metabolites in pFC and AMY. Serotonergic metabolism resulted reduced also in CgC. This data suggest that moderately high PHE levels, typically reported after discontinuation of dietary management of PKU during adolescence, produce brain metabolic alterations in particular in 5-HT and NE levels in cortical areas and AMY. Cognitive and emotional disturbances reported in patients with moderate HPA may be related to these metabolic deficits [2].

It should be noted that, although previous studies accidentally reported in m-HPA mice higher blood PHE levels in comparison with wild-type [13,35], this data did not receive special attention. Rather, recently heterozygous Pah^Enu2^ mice have been often used as control group instead of wild-type subjects, so abolishing the costs of genetic characterization following heterozygous mating. Present data confirm hyperphenylalaninemic status of heterozygous Pah^Enu2^ mice, previously reported, and provide behavioral and neurochemical characterization that questions about the use of heterozygous Pah^Enu2^ as control group of phenylketonuric mice.

In conclusion, our study reports behavioral and neurochemical characterization of a murine model modeling different degrees of HPA. Progressive increase of PHE blood levels produced increasing severity of behavioral symptoms, related to graduate impairment of aminergic metabolism in several brain areas. Moreover, our study confirms the highest susceptibility of brain 5-HT metabolism to blood PHE levels. Finally, the HPA mice models proposed here offer a valuable tool to investigate the relationship between PHE-dependent behavioral and brain deficits, and for the preclinical assessment of therapeutic approach for specific HPA forms.

## References

[1] Santos LL, Magalhães MC, Januário JN, Aguiar MJ, Carvalho MR (2006) The time has come: a new scene for PKU treatment. Genet Mol Res 5: 33-44. PubMed: 16755495. 16755495

[2] Brumm VL, Azen C, Moats RA, Stern AM, Broomand C et al. (2004) Neuropsychological outcome of subjects participating in the PKU Adult Collaborative Study: A preliminary review. J Inherit Metab Dis 27: 549-566. doi:10.1023/B:BOLI.0000042985.02049.ff. PubMed: 15669671. 15669671

[3] Channon S, German C, Cassina C, Lee P (2004) Executive functioning, memory, and learning in phenylketonuria. Neuropsychology 18: 613-620. doi:10.1037/0894-4105.18.4.613. PubMed: 15506828. 15506828

[4] Diamond A, Prevor MB, Callender G, Druin D (1997) Prefrontal cortex cognitive deficits in children treated early and continuously for PKU. Monogr Soc Res Child Dev 62: 1-208. PubMed: 9353949. 9421921

[5] Huijbregts SCJ, de Sonneville LMJ, Licht R, van Spronsen FJ, Verkerk PH et al. (2002) Sustained attention and inhibition of cognitive interference in treated phenylketonuria: Associations with concurrent and lifetime phenylalanine concentrations. Neuropsychologia 40: 7-15. doi:10.1016/S0028-3932(01)00078-1. PubMed: 11595258. 11595258

[6] Leuzzi V, Pansini M, Sechi E, Chiarotti F, Carducci C et al. (2004) Executive function impairment in early-treated PKU subjects with normal mental development. J Inherit Metab Dis 27: 115-125. doi:10.1023/B:BOLI.0000028781.94251.1f. PubMed: 15159642. 15159642

[7] Schmidt E, Rupp A, Burgard P, Pietz J, Weglage J et al. (1994) Sustained attention in adult phenylketonuria: the influence of the concurrent phenylalanine-blood-level. J Clin Exp Neuropsychol 16: 681-688.. 10.1080/01688639408402681 7836491

[8] Smith ML, Hanley WB, Clarke JT, Klim P, Schoonheyt W et al. (1998) Randomised controlled trial of tyrosine supplementation on neuropsychological performance in phenylketonuria. Arch Dis Child 78: 116–121. doi:10.1136/fn.78.2.F116. PubMed: 9579151. 9579151 PMC1717450

[9] White D, Nortz M, Mandernach T, Huntington K, Steiner R (2002). Available: Age-related working memory impairments in children with prefrontal dysfunction associated with phenylketonuria. J Int Neuropsychol Soc 8: 1-11.. 11843066

[10] McDonald JD, Bode VC, Dove WF, Shedlovsky A (1990). Available: Pahhph-5: a mouse mutant deficient in phenylalanine hydroxylase. Proc Natl Acad Sci USA 87: 1965-1967.. 2308957 10.1073/pnas.87.5.1965 PMC53605

[11] Shedlovsky A, McDonald JD, Symula D, Dove WF (1993) Mouse model of phenylketonuria. Genetics 134: 1205-1210. PubMed: 8375656. 8375656 10.1093/genetics/134.4.1205 PMC1205587

[12] Joseph B, Dyer CA (2003) Relationship between myelin production and dopamine synthesis in the PKU mouse brain. J Neurochem; 86: 615–626. doi:10.1046/j.1471-4159.2003.01887.x. PubMed: 12859675. 12859675

[13] Cabib S, Pascucci T, Ventura R, Romano V, Puglisi-Allegra S (2003) The behavioral profile of severe mental retardation in a genetic mouse model of phenylketonuria. Behav Genet 33: 301-310. doi:10.1023/A:1023498508987. PubMed: 12837019. 12837019

[14] Zagreda L, Goodman J, Druin DP, McDonald D, Diamond A (1999) Cognitive deficits in a genetic mouse model of the most common biochemical cause of human mental retardation. J Neurosci 19: 6175–6182. PubMed: 10407053. 10407053 10.1523/JNEUROSCI.19-14-06175.1999 PMC6783085

[15] Andolina D, Conversi D, Cabib S, Trabalza A, Ventura R et al. (2011) 5-Hydroxytryptophan during critical postnatal period improves cognitive performances and promotes dendritic spine maturation in genetic mouse model of phenylketonuria. Int J Neuropsychopharmacol 14: 479-489. doi:10.1017/S1461145710001288. PubMed: 21040618. 21040618 PMC3110346

[16] Pascucci T, Ventura R, Puglisi-Allegra S, Cabib S (2002) Deficits in brain serotonin synthesis in a genetic mouse model of phenylketonuria. Neuroreport 13: 2561-2564. doi:10.1097/00001756-200212200-00036. PubMed: 12499868. 12499868

[17] Pascucci T, Andolina D, Ventura R, Puglisi-Allegra Cabib S (2008) Reduced availability of brain amines during critical phases of postnatal development in a genetic mouse model of cognitive delay. Brain Res 1217: 232-238. doi:10.1016/j.brainres.2008.04.006. PubMed: 18502400. 18502400

[18] Pascucci T, Andolina D, La Mela I, Conversi D, Latagliata C et al. (2009) 5-Hydroxytryptophan rescues serotonin response to stress in prefrontal cortex of hyperphenylalaninaemic mice. Int J Neuropsychopharmacol 12: 1067-1079. doi:10.1017/S1461145709990381. PubMed: 19664307. 19664307

[19] Pascucci T, Giacovazzo G, Andolina D, Conversi D, Cruciani F et al. (2012) In vivo catecholaminergic metabolism in the medial prefrontal cortex of ENU2 mice: an investigation of the cortical dopamine deficit in phenylketonuria. J Inherit Metab Dis 35: 1001-1009. doi:10.1007/s10545-012-9473-2. PubMed: 22447154. 22447154 PMC3470696

[20] Puglisi-Allegra S, Cabib S, Pascucci T, Ventura R, Cali F et al. (2000) Dramatic brain aminergic deficits in a genetic mouse model of phenylketonuria. Neuroreport 11: 1361-1364. doi:10.1097/00001756-200004270-00042. PubMed: 10817622. 10817622

[21] Chace DH, Millington DS, Terada N, Kahler SG, Roe CR et al. (1993) Rapid diagnosis of phenylketonuria by quantitative analysis for phenylalanine and tyrosine in neonatal blood spots by tandem mass spectrometry. Clin Chem 39: 66-71. PubMed: 8419060. 8419060

[22] Van Haaren F, De Bruin JP, Heinsbroek RP, Van de Poll NE (1985) Delayed spatial response alternation: effects of delay-interval duration and lesions of the medial prefrontal cortex on response accuracy of male and female Wistar rats. Behav Brain Res 18: 41–49. doi:10.1016/0166-4328(85)90167-6. PubMed: 4091955. 4091955

[23] Moran PM (1993) Differential effects of scopolamine and mecamylamine on working and reference memory in the rats. Pharmacol Biochem Behav 45: 533–853. doi:10.1016/0091-3057(93)90502-K. PubMed: 8332613. 8332613

[24] Zahrt J, Taylor JR, Mathew RG, Arnsten AFT (1997) Supranormal stimulation of D1 dopamine receptors in the rodent prefrontal cortex impairs spatial working memory performance. J Neurosci 17: 8528–8535. PubMed: 9334425. 9334425 10.1523/JNEUROSCI.17-21-08528.1997 PMC6573725

[25] Mizoguchi K, Yuzurihara M, Ishige A, Sasaki H, Chui DH et al. (2000) Chronic stress induces impairment of spatial working memory because of prefrontal dopaminergic dysfunction. J Neurosci 20: 1568–1574. PubMed: 10662846. 10662846 10.1523/JNEUROSCI.20-04-01568.2000 PMC6772382

[26] de Groot MJ, Hoeksma M, Blau N, Reijngoud DJ, van Spronsen FJ (2010) Pathogenesis of cognitive dysfunction in phenylketonuria: Review of hypotheses. Mol Genet Metab 99: S86-S89. doi:10.1016/j.ymgme.2009.10.016. PubMed: 20123477. 20123477

[27] Martynyuk AE, van Spronsen FJ, Van der Zee EA (2010) Animal models of brain dysfunction in phenylketonuria. Mol Genet Metab 99: S100-S105. doi:10.1016/j.ymgme.2009.10.181. PubMed: 20123463. 20123463

[28] Brumm VL, Bilder D, Waisbren SE (2010) Psychiatric symptoms and disorders in phenylketonuria. Mol Genet Metab 99: S59-S63. doi:10.1016/j.ymgme.2009.10.182. PubMed: 20123472. 20123472

[29] De Roche K, Welsh MC (2008) Twenty-five years of research on neurocognitive outcomes in early-treated phenylketonuria: intelligence and executive function. Dev Neuropsy 33: 474-504. doi:10.1080/87565640802101482. 18568900

[30] Stemerdink BA, Kalverboer AF, van der Meere JJ, van der Molen MW, Huisman J et al. (2000) Behavioural and school achievement in patients with early and continuously treated phenylketonuria. J Inherit Metab Dis 23: 548-562. doi:10.1023/A:1005669610722. PubMed: 11032330. 11032330

[31] McKean CM (1972) The effects of high phenylalanine concentrations on serotonin and catecholamine metabolism in the human brain. Brain Res 47: 469-476. doi:10.1016/0006-8993(72)90653-1. PubMed: 4642573. 4642573

[32] Burlina AB, Bonafé L, Ferrari V, Suppiej A, Zacchello F et al. (2000) Measurement of neurotransmitter metabolites in the cerebrospinal fluid of phenylketonuric patients under dietary treatment. J Inherit Metab Dis 23: 313-316. doi:10.1023/A:1005694122277. PubMed: 10896282. 10896282

[33] Butler IJ, O’Flynn ME, Seifert WE Jr, Howell RR (1981) Neurotransmitter defects and treatment of disorders of hyperphenylalaninemia. J Pediatr 98: 729-733. doi:10.1016/S0022-3476(81)80832-3. PubMed: 6112253. 6112253

[34] Lou H (1985) Large doses of tryptophan and tyrosine as potential therapeutic alternative to dietary phenylalanine restriction in phenylketonuria. Lancet 20: 150–151. PubMed: 2862338. 10.1016/s0140-6736(85)90250-8 2862338

[35] Smith CB, Kang J (2000) Cerebral protein synthesis in a genetic mouse model of phenylketonuria. Proc Natl Acad Sci U S A; 97: 11014-11019. doi:10.1073/pnas.97.20.11014. PubMed: 11005872. 11005872 PMC27140

